# Oculomotor evaluation in patients with peripheral vestibular dysfunction

**DOI:** 10.1016/S1808-8694(15)30977-0

**Published:** 2015-10-19

**Authors:** Vanessa Costa Tuma, Cristina Freitas Ganança, Maurício Malavasi Ganança, Heloisa Helena Caovilla

**Affiliations:** a Master in Sciences - Post-graduate program on Human Communication Disorders: Phonoaudiological Area - Unifesp-EPM, phonoaudiologist; b Master in Sciences - Post-graduate program on Human Communication Disorders: Phonoaudiological Area - Unifesp-EPM, phonoaudiologist; c Full Professor of Otorhinolaryngology at Unifesp-EPM; d Associate Professor of Otoneurology at Unifesp-EPM. Sao Paulo Federal University - Paulista School of Medicine

**Keywords:** vestibular dysfunction, vectonystagmography, vertigo

## Abstract

**Aim:**

To assess whether or not the parameters of fixed and randomized saccadic movements, of pendular tracking and of optokinetic nystagmus in the digital vectonystagmography may show abnormalities in patients with possible diagnosis of peripheral vestibular dysfunction.

**Method:**

60 patients with dizziness of peripheral vestibular origin, from 12 to 82 years of age, males and females, were evaluated in the Universidade Federal de São Paulo. Ocular movement parameter findings were compared to a normal pattern.

**Results:**

Fixed saccadic movements were altered in 100% of the cases as to latency, and in 35.0% of the cases as to speed; the randomized saccadic movements were altered in 100% of the cases as to latency, in 78.3% as to precision, and in 1.7% as to speed; the pendular tracking showed a gain alteration in the frequencies of 0.1 Hz in 15% of the cases, 0.2 Hz in 21.7%, and 0.4 Hz in 13.3%; the optokinetic nystagmus showed an alteration of the angular speed in the slow component in 1.7% of the cases, and in gain in 5.0%.

**Conclusion:**

Fixed saccadic movement latency and speed, randomized saccadic movement latency, precision and speed, pendular tracking gain, slow component angular speed, and optokinetic nystagmus gain in the digital vectonystagmography may show abnormalities in patients with possible diagnosis of peripheral vestibular dysfunction.

## INTRODUCTION

Balance depends on visual, proprioceptive and vestibular sensorial information that establish a physiological pattern recognized by the central nervous system (CNS). Integration of such information takes place in the vestibular nuclei within the brain stem. The CNS processes and organizes sensorial information and is responsible for motor planning and control, setting in motion adequate ocular and spinal reflexes to automatically and unconsciously maintain balance and static and dynamic spatial orientation in the environment^1^.

Functional examination of the vestibular system may be done using electronystagmography or vectonystagmography, which investigate possible vestibular involvement, identify the affected side, establish the topodiagnosis of the lesion (peripheral or central), characterize the type of injury, define the prognosis and monitor the clinical progression after treatment is started^2^.

Digital vectonystagmography is frequently used in our setting to assess vestibular function, allowing greater diagnosis sensitivity by measuring vestibular-oculomotor function parameters, comparing stimulus and response, and identifying the direction of ocular phenomena^3^.

Five eye movement systems have been described: smooth pursuit, which keeps the image of a moving object in the fovea; saccadic, which positions the image of a target over the fovea; vestibular, which generates eye movements equal and opposite to those of the head; optokinetic, which generates slow pursuit movements and rapid refixation movements in response to image movements; and vergence, which is responsible for eye movements in opposite directions to position the image in both foveae^4^.

The saccadic pathway involves many regions of the brain cortex, the cerebellum and the brain stem. Fixed or randomized saccadic movement latency, velocity and accuracy assess the efficiency of CNS control on rapid eye movements^5^. Few disorders affecting the CNS are undetected when saccadic movement latency, velocity and accuracy are measured precisely by means of a computer^6^.

Slow pursuit movements stabilize the image of a target that moves over the fovea. This system is extremely vulnerable to CNS dysfunction and frequently causes clinical symptoms^7^. The velocity of slow pursuit movements may be considered a sensitive measure of brain stem dysfunction^8^.

Pendular tracking is the resulting eye movement from following a moving target and may assess the integrity of the oculomotor system in controlling slow ocular movements, which are vulnerable to CNS and vestibular system dysfunction^1^.

Type I and II pendular tracking are found in normal persons and in patients with diseases of the labyrinth. Types III and IV are found in degenerative cerebellar disorders, vascular occlusion of cerebellar arteries, pontocerebellar angle tumors, congenital nystagmus, hypertension and diabetes with advanced vasculopathy^9^.

Optokinetic nystagmus is a rhythmic, involuntary, unconscious and automatic ocular phenomenon. It may be reproduced by following points of light that move first in one direction and then in the opposite direction. It is an exteroceptive response that compensates movements in the environment by psycho-optic impulses. Optokinetic nystagmus may be altered in CNS syndromes and in vestibular dysfunction^1^.

The aim of this study was to define whether fixed and randomized saccadic movement, pendular tracking and optokinetic nystagmus parameters were altered on digital vectonystagmography in patients with a diagnostic hypothesis of peripheral vestibular dysfunction.

## METHODS

Sixty peripheral vestibular dysfunction patients from the Balance Measurement Unit of the Otoneurology Department at Sao Paulo Federal University (Paulista School of Medicine) in Sao Paulo underwent digital vectonystagmography to assess fixed and randomized saccadic movement, pendular tracking and optokinetic nystagmus oculomotor parameters.

The research protocol was approved by the Research Ethics Committee of the abovementioned institution; participants read and signed a free and informed consent form.

Patients enrolled in the study were aged between 12 and 82 years; 19 (31.7%) were male and 41 (68.3%) were female. Patients were instructed not to take drugs for dizziness, tranquilizers or sedatives 72 hours before, and not to drink tea, coffee, chocolate, soft drinks, alcoholic beverages or to smoke 48 hours before vestibular function tests, to avoid interference with eye movements, consequently affecting test results^2^. On the day of the examination, patients were asked to take light meals and to fast three hours before testing.

Electronystagmography procedures adhered to positioning nystagmus investigation guidelines; calibration of eye movements; investigation of spontaneous, semi-spontaneous and optokinetic nystagmus; fixed and randomized saccadic movements; pendular tracking; rotational chair testing and caloric test with air^2^. A VECWIN digital vectonystagmograph, a light bar and a NGR-05 air caloric stimulator (Neurograff Eletromedicina Ind. e Com. Ltda-EPT) were used for testing.

Positioning nystagmus was investigated by seating the patient, placing the head at a 45 degree angle to one side, rapidly reclining the patient to lateral decubitus on the opposite side, maintaining the position, then rapidly moving the patient back to the seated position, and then repeating the maneuver with the head angled 45 degrees to the other side. Each position was maintained for 30 seconds or until dizziness and/or nystagmus yielded or disappeared. Before the test patients were instructed not to oppose motion and not to close their eyes.

Skin was cleaned and three active electrodes and one ground electrode were placed before digital vectonystagmography. Three derivation^10^ active electrodes were placed on the right and left external periorbitary angles and on the frontal midline to record eye movements in three recording channels.

Calibration was done to assure similar conditions for the various steps of the test and to automatically record latency, precision, velocity and gain for eye movements other than the slow component of nystagmus.

Spontaneous nystagmus was investigated in central gaze with open and closed eyes. Semi-spontaneous nystagmus was investigated in right, left, upward and downward gaze deviation not more than 30 degrees from the midline. The slow component velocity was measured during the test.

Saccadic movements were assessed by visually following a regularly or randomly moving target. Saccade latency, velocity and precision were assessed.

Pendular tracking was assessed by having the patient visually follow the sinusoidal movement of a light target at 0.2 and 0.4 Hz frequency. Eye movement type and gain were assessed in this test.

Optokinetic nystagmus gain and velocity were measured by having the patient visually follow a moving light target with a fixed direction to one and to the other side at 10°/s.

Rotational chair testing was done with the patients seated, eyes closed and the head tilted forward at a 30 degree angle to stimulate lateral semicircular canals, tilted backwards at a 60 degree angle to stimulate posterior and superior semicircular canals, then tilted 45 degrees to the right, then 60 degrees backwards and 45 degrees to the left. The chair was rotated 90 degrees from center and released, resulting in a decreasing amplitude periodic pendular movement. Resulting peri-rotational nystagmus was assessed by measuring the velocity of its slow component. Pre-rotational nystagmus and its influence on test results were also investigated.

Caloric testing was done stimulating each ear separately with air at 18°C and 42°C during 80 seconds. Post-caloric vertigo and the direction and velocity of the slow component of nystagmus were assessed with open and closed eyes. Pre-caloric nystagmus and its influence on test results were also investigated.

These tests were used to define peripheral vestibular dysfunction. Fixed and randomized saccadic movements, pendular tracking and optokinetic nystagmus were evaluated and compared to normal limits^11^, using the same equipment and procedures. Statistical analysis included quantitative variables such as the average, the mean, standard deviation (SD), minimal and maximal values, and upper and lower limits; qualitative variables were absolute frequency (n) and relative frequency (%). Analysis of variance (ANOVA) was used to compare right and left movements in fixed and randomized saccadic tests and optokinetic nystagmus. As no statistically significant difference was found in these evaluations, the units were named “eye movement”. ANOVA was used to compare the averages in this study with normal limits. The Equality of Two Proportions test was used to compare the proportion of responses to two specific variables and/or its levels and if these were statistically significant. The Confidence Interval test was used to verify how much the average could vary in a specific confidence probability. A 0.05 (∝ = 5%) significance level was used and descriptive levels (p) below this value were considered significant and a represented by an asterisk (*).

## RESULTS

The study assessed oculomotor tests in digital vectonystagmography of 60 patients with peripheral vestibular dizziness.

Fixed saccadic movements were considered regular for all cases.

Table 1 shows that there was no statistically significant difference between right and left eye movements, considering latency (in ms), velocity (in º/s) and precision of fixed saccadic movements.

**Table 1:** Comparison of fixed saccadic movement latency, velocity and precision values to the left and to the right.

Fixed Saccadic Movements		To the left			To the right	
	Latency	Velocity	Precision	Latency	Velocity	Precision
Average	203,99	144,14	104,47	198,75	144,70	103,74
Mean	195,25	142,90	104,60	191,40	146,35	103,75
Standard deviation	57,88	19,89	9,96	56,95	23,96	8,75
Minimum value	99,60	102,40	73,60	95,70	60,50	74,60
Maximum value	335,90	199,90	142,60	330,00	196,30	128,30
Lower limit	189,34	139,11	101,95	184,34	138,63	101,53
Upper limit	218,63	149,17	106,99	213,16	150,76	105,95

Table 2 shows that there was no statistically significant difference between right and left eye movements, considering latency (in ms), velocity (in º/s) and precision of randomized saccadic movements.

**Table 2:** Comparison of randomized saccadic movement latency, velocity and precision values to the left and to the right.

Randomized Saccadic Movements		To the left			To the right	
	Latency	Velocity	Precision	Latency	Velocity	Precision
Average	206,62	96,39	100,64	197,99	84,05	102,24
Mean	200,15	95,50	102,10	192,35	83,70	101,95
Standard deviation	53,69	20,05	13,85	40,00	13,81	15,26
Minimum value	119,10	57,60	73,80	138,60	57,30	69,90
Maximum value	357,40	147,10	139,30	296,80	119,80	152,50
Lower limit	193,04	91,31	97,13	187,87	80,55	98,38
Upper limit	220,21	101,46	104,14	208,11	87,54	106,10

Table 3 shows that there was no statistically significant difference between right and left eye movements, considering gain and angular velocity of the slow component of optokinetic nystagmus.

**Table 3:** Comparison of slow component angular velocity (SCV) values and optokinetic nystagmus gain to the left and to the right.

Optokinetic nystagmus	SCV p/E	SCV p/D	GAIN		PDN %
			p/E	p/D	
Average	10,03	10,11	1,00	1,01	3,93
Mean	10,05	10,00	0,88	0,84	3,20
Standard deviation	1,76	2,23	0,99	1,14	3,46
Minimum value	6,10	5,50	0,59	0,53	0,00
Maximum value	13,70	20,60	8,40	9,60	13,00
Lower limit	9,58	9,55	0,75	0,72	3,05
Upper limit	10,48	10,67	1,25	1,30	4,80

Legend:

SCV = slow component angular velocity

p/D = optokinetic nystagmus to the right

p/E = optokinetic nystagmus to the left

Table 4 shows statistical data on latency, velocity and precision values of fixed saccadic movements.

**Table 4:** Statistical data on fixed saccadic movement latency, velocity and precision values.

Fixed Saccadic Movements	Latency	Velocity	Precision
Average	201,37	144,42	104,10
Mean	192,35	144,70	104,10
Standard deviation	57,24	21,93	9,34
Minimum value	95,70	60,50	73,60
Maximum value	335,90	199,90	142,60
Lower limit	191,13	140,49	102,43
Upper limit	211,61	148,34	105,77

Table 5 shows statistical data on latency, velocity and precision values of randomized saccadic movements.

**Table 5:** Statistical data on randomized saccadic movement latency, velocity and precision values.

Randomized Saccadic Movements	Latency	Velocity	Precision
Average	202,31	90,22	101,45
Mean	195,25	87,90	102,00
Standard deviation	47,34	18,23	14,54
Minimum value	119,10	57,30	69,90
Maximum value	357,40	147,10	152,50
Lower limit	193,84	86,95	98,84
Upper limit	210,78	93,48	104,05

Table 6 shows statistical data on angular velocity of the slow component values and gain of optokinetic nystagmus.

**Table 6:** Statistical data on slow component angular velocity and optokinetic nystagmus gain values.

Optokinetic Nystagmus	SCV	GAIN
Average	10,07	1,00
Mean	10,00	0,86
Standard deviation	2,00	1,06
Minimum value	5,50	0,53
Maximum value	20,60	9,60
Lower limit	9,71	0,81
Upper limit	10,43	1,19

Table 7 shows statistical data on gain values in pendular tracking at 0.1, 0.2 and 0.4Hz frequencies.

**Table 7:** Statistical data on pendular tracking gain values at frequencies of 0.1, 0.2 and 0.4Hz.

Pendular tracking	GAIN		
	0,1Hz	0,2Hz	0,4Hz
Average	0,76	0,91	0,93
Mean	0,74	0,91	0,94
Standard deviation	0,18	0,15	0,16
Minimum value	0,33	0,60	0,54
Maximum value	1,21	1,21	1,28
Lower limit	0,71	0,87	0,89
Limite Superior	0,80	0,94	0,97

Table 8 shows that all patients (100.0%) had altered fixed saccadic movement latency and 35.0% of patients had an altered velocity. Precision was within normal limits in all cases (100.0%). A higher proportion of patients had altered latency results and a higher proportion of patients with results within normal limits for velocity and precision. The difference between normal and altered results was statistically significant.

**Table 8:** Distribution of patients with altered and normal fixed saccadic movement latency, velocity and precision.

Fixed Saccadic Movements	Normal		Altered		p-value
	N	%	N	%	
Latency	0	0,0	60	100,0	< 0,001*
Velocity	39	65,0	21	35,0	0,001*
Precision	60	100,0	0	0,0	< 0,001*

Legend

N = number of cases

Table 9 shows that for randomized saccadic movements, all cases (100.0%) had altered latency, 1.7% of cases had altered velocity and 78.3% had altered precision. A higher proportion of patients had altered latency and precision results and a higher proportion of patients with results within normal limits for velocity. The difference between normal and altered results for the three parameters was statistically significant.

**Table 9:** Distribution of patients with altered and normal randomized saccadic movement latency, velocity and precision.

Randomized Saccadic Movements	Normal		Altered		p-value
	N	%	N	%	
Latency	0	0,0	60	100,0	< 0,001*
Velocity	59	98,3	1	1,7	< 0,001*
Precision	13	21,7	47	78,3	< 0,001*

N = number of cases

Optokinetic nystagmus was symmetrical in the comparison for both directions in all cases.

Table 10 shows that 1.7% of cases had altered slow component angular velocities and 50.% had gains in optokinetic nystagmus. The directional predominance of optokinetic nystagmus was within normal limits in all cases. A higher proportion of patients had slow component angular velocities and gain within normal limits. The difference between normal and altered results for both parameters was statistically significant.

**Table 10:** Distribution of patients with altered and normal slow component angular velocity and optokinetic nystagmus gain.

Optokinetic nystagmus	Normal		Altered		p-value
	N	%	N	%	

SCV	59	98,3	1	1,7	< 0,001*
Gain	57	95,0	3	5,0	< 0,001*

Legend

SCV = slow component angular velocity

N = number of cases

Normal results were found in all cases for pendular tracking types I or II.

Table 11 shows that 15.0% of cases had altered frequency gain at 0.1Hz, 21.7% had altered frequency gain at 0.2Hz and 13.3% had altered frequency gain at 0.4Hz. A higher proportion of patients had results within normal limits in all frequencies. The difference between normal and altered results for all three frequencies was statistically significant.

**Table 11:** Distribution of patients with altered and normal pendular tracking gain at frequencies of 0.1, 0.2 and 0.4Hz.

Pendular tracking	Normal		Altered		p-value
	N	%	N	%	

0,1Hz	51	85,0	9	15,0	< 0,001*
0,2Hz	47	78,3	13	21,7	< 0,001*
0,4Hz	52	86,7	8	13,3	< 0,001*

Legend

N = number of cases

## DISCUSSION

Regular fixed saccadic movement tracings were seen for all patients with peripheral vestibular dysfunction, in accordance with literature^12^.

A higher proportion of cases had altered fixed saccadic movement latency and normal velocity and precision. The difference between normal and altered results for the three parameters was statistically significant. Reports^13^ demonstrate that for fixed saccadic movements, 36.0% of children with migraine had altered latency, 39.1% of children with migraine had altered velocity and 26.1% of children with migraine had altered precision.

A higher proportion of cases had altered randomized saccadic movement latency and precision as well as cases with normal velocity. The difference between normal and altered results for the three parameters was statistically significant. Reports^13^ demonstrate that for randomized saccadic movements, 13.0% of children with migraine had altered latency, 34.8% of children with migraine had altered velocity and 17.4% of children with migraine had altered precision, different from our results.

We found no reference in literature about statistical assessments on fixed and randomized saccadic movements to compare with our findings.

Analyzing saccadic movements in peripheral vestibular dysfunction, literature indicates altered latency in a significant number of cases^6^, ^7^, ^8^, ^9^, ^10^, ^11^, ^12^, ^13^, ^14^ or in few cases^16^. Altered velocity was found in 13.0% of cases and altered precision was found in 3% of cases^15^. The high prevalence of altered fixed and randomized saccadic movement latency and altered velocity and precision seen in this study in peripheral vestibular dysfunction may be a sign of vestibule-oculomotor dysfunction not necessarily located in the CNS^3^.

Optokinetic nystagmus testing usually reveals slow component angular velocity symmetry in normal subjects^12^ or with peripheral vestibular dysfunction^16^. Increased slow component angular velocity is seen in 8.7% of children with migraine^13^, 4.3% of children with migraine have altered gain and 21.7% of children with migraine have directional predominance of optokinetic nystagmus. We found a higher proportion of cases with no optokinetic nystagmus velocity and gain abnormalities. The difference between normal and altered results for both parameters was statistically significant. No references on statistical assessments on optokinetic nystagmus were found to allow comparisons with our findings.

The pendular tracking test showed type I or II tracings^12^, ^13^, ^17^, ^18^, ^19^. Altered gain in pendular tracking may occur in peripheral vestibular dysfunction^13^, ^16^. In our study we found a higher proportion of cases with no pendular tracking gain abnormalities. The difference between normal and altered results for this parameter was statistically significant. No references on statistical assessments on pendular tracking were found to allow comparisons with our findings.

From what we saw in this study, altered parameters found in fixed and randomized saccadic movements, optokinetic nystagmus and pendular tracking on digital vectonystagmography may also be found in patients with peripheral vestibular dizziness. Therefore, caution is needed when considering these oculomotor changes as measures of brain stem dysfunction^1^, ^7^, ^8^. Further studies are needed to understand the topodiagnostic implications of qualitative and quantitative aspects of these changes in peripheral and central vestibular dysfunction.

## CONCLUSION

Fixed saccadic movement latency and velocity, randomized saccadic movement latency, precision and velocity, pendular tracking gain, slow component angular velocity and optokinetic nystagmus gain on digital vectonystagmography may be altered in patients with a diagnostic hypothesis of peripheral vestibular dysfunction.
